# Health-Related Quality of Life and Cost-Effectiveness of First-Line Epidermal Growth Factor Receptor (EGFR) Tyrosine Kinase Inhibitor Monotherapy in Advanced EGFR-Mutated Non-small Cell Lung Cancer: A Systematic Review

**DOI:** 10.7759/cureus.115032

**Published:** 2026-08-23

**Authors:** Subodh Kumar, Shiv K Mudgal, Seshadri Reddy Varikasuvu, Dharmendra Singh, Harminder Singh, Sumit Mahato, Pradosh Kumar Sarangi, Himel Mondal

**Affiliations:** 1 Department of Pharmacology, All India Institute of Medical Sciences, Deoghar, IND; 2 Department of Evidence Synthesis (DOES), All India Institute of Medical Sciences, Deoghar, IND; 3 College of Nursing, Dr. Ram Manohar Lohia Institute of Medical Sciences, Lucknow, IND; 4 Department of Biochemistry, All India Institute of Medical Sciences, Deoghar, IND; 5 Department of Radiation Oncology, All India Institute of Medical Sciences, Deoghar, IND; 6 Department of Radiodiagnosis, All India Institute of Medical Sciences, Deoghar, IND; 7 Department of Physiology, All India Institute of Medical Sciences, Deoghar, IND

**Keywords:** cost-effectiveness, economic evaluation, egfr-mutated nsclc, egfr tyrosine kinase inhibitors, first-line therapy, health-related quality of life, non-small cell lung cancer, patient-reported outcomes, precision oncology, systematic review

## Abstract

First-line epidermal growth factor receptor tyrosine kinase inhibitors (EGFR-TKIs) have transformed the management of advanced EGFR-mutated non-small cell lung cancer (NSCLC). Although randomized trials have demonstrated improvements in clinical outcomes, treatment decisions increasingly require consideration of health-related quality of life (HRQoL) and economic value. This systematic review synthesized evidence on HRQoL and cost-effectiveness of first-line EGFR-TKI therapies in advanced EGFR-mutated NSCLC. This systematic review was conducted in accordance with the Preferred Reporting Items for Systematic Reviews and Meta-Analyses 2020 statement and prospectively registered in PROSPERO (CRD42024553180). PubMed/MEDLINE, Embase, Scopus, and the Cochrane Library were searched from database inception to 31 March 2026. Eligible publications included randomized controlled trial reports assessing HRQoL using validated patient-reported outcome instruments and full trial-informed economic evaluations of first-line EGFR-TKI strategies. Risk of bias in HRQoL studies was assessed using the Cochrane Risk of Bias 2 tool, whereas the methodological quality of economic evaluations was assessed using the Drummond checklist. Owing to methodological heterogeneity, findings were synthesized narratively. A total of nine publications linked to six pivotal randomized controlled trials met the inclusion criteria, comprising four HRQoL reports and five trial-informed economic evaluations. Across the included studies, global health status and functional outcomes were generally maintained or improved from baseline, and no consistent clinically meaningful between-group differences were demonstrated; however, the limited evidence base does not establish equivalence between individual EGFR-TKIs. Economic findings varied across healthcare settings. Afatinib and aumolertinib were considered cost-effective within the settings examined, whereas osimertinib, dacomitinib, and lazertinib exceeded the willingness-to-pay thresholds used in the respective analyses. Cost-effectiveness was influenced by drug acquisition costs, model assumptions, analytical perspectives, and country-specific thresholds. Available randomized-trial evidence suggests that global HRQoL does not clearly differentiate the currently evaluated first-line EGFR-TKIs, as most treatments maintained overall health status and functioning despite differences in efficacy and toxicity. However, these findings should be interpreted cautiously given the limited number and heterogeneity of available HRQoL studies. In contrast, cost-effectiveness varied markedly across healthcare systems and appeared to be largely driven by drug acquisition costs and country-specific willingness-to-pay thresholds. The principal value distinction among first-line EGFR-TKIs may therefore lie less in global HRQoL and more in whether additional clinical benefit is considered affordable within the local healthcare setting.

## Introduction and background

Lung cancer remains the leading cause of cancer-related mortality worldwide. In 2022, it accounted for approximately 2.5 million new cases and 1.8 million deaths, representing 12.4% of all newly diagnosed cancers and 18.7% of cancer deaths globally [1]. Non-small cell lung cancer (NSCLC) constitutes the majority of lung cancers. The identification of actionable oncogenic alterations has transformed the management of advanced NSCLC by enabling the selection of targeted therapies according to tumor molecular characteristics. Among these alterations, activating mutations in the epidermal growth factor receptor (EGFR) are particularly important and occur more frequently in Asian than in Western populations [2].

EGFR tyrosine kinase inhibitors (EGFR-TKIs) have become a cornerstone of first-line treatment for patients with advanced EGFR-mutated NSCLC. First-generation agents, including gefitinib and erlotinib, established the clinical value of EGFR-targeted therapy, while second-generation agents, such as afatinib and dacomitinib, provided broader and irreversible inhibition of the ErbB receptor family. More recently, third-generation EGFR-TKIs, including osimertinib, furmonertinib, aumolertinib, and lazertinib, have been developed to improve selectivity for mutant EGFR, central nervous system activity, and activity against resistance-associated mutations. Randomized trials have demonstrated differences among individual EGFR-TKIs in progression-free survival, overall survival, toxicity, and patient-reported outcomes [3-8], while network meta-analyses have compared these outcomes across a broader range of first-line strategies [9-11]. In the FLAURA trial, osimertinib improved both progression-free and overall survival compared with gefitinib or erlotinib [3,4]. Further, the first-line treatment has expanded to include combination approaches, including osimertinib plus chemotherapy and EGFR-directed antibody/TKI combinations. These strategies were outside the prespecified scope of the present review, which focused on first-line EGFR-TKI monotherapy.

As survival improves and treatment continues for prolonged periods, preservation of health-related quality of life (HRQoL) has become an increasingly important therapeutic objective. HRQoL reflects the effects of disease and treatment on patients’ physical, emotional, social, and functional well-being and complements conventional clinical outcomes such as tumor response and survival. Although EGFR-TKIs are generally more tolerable than conventional chemotherapy, prolonged exposure may produce chronic adverse effects, including diarrhea, rash, paronychia, stomatitis, and fatigue, which may influence daily functioning, adherence, and treatment acceptability. Patient-reported outcomes from major randomized trials have therefore become increasingly relevant when evaluating the overall clinical value of first-line EGFR-TKIs [5-8].

The increasing availability of multiple effective EGFR-TKIs has also raised concerns regarding affordability and efficient healthcare-resource allocation. Newer agents may provide additional clinical benefit but are often associated with substantially higher acquisition costs. Economic evaluations use measures such as quality-adjusted life-years (QALYs), incremental costs, incremental cost-effectiveness ratios (ICERs), and willingness-to-pay (WTP) thresholds to assess whether additional health benefits justify the associated expenditure. However, cost-effectiveness conclusions may differ considerably across healthcare systems because of variations in drug prices, analytical perspectives, healthcare-resource use, model assumptions, and country-specific thresholds [12].

HRQoL and cost-effectiveness represent distinct but complementary dimensions of treatment value. HRQoL captures the patient’s experience of treatment and disease control, whereas economic evaluation examines whether the resulting health benefits represent acceptable value within a particular healthcare system. Although economic evaluations may incorporate utility estimates and QALYs, these modelled outcomes are not equivalent to the detailed patient-reported HRQoL findings collected prospectively in randomized trials. Nevertheless, because economic evaluations of first-line EGFR-TKIs are commonly informed by efficacy and survival data from pivotal randomized trials, integrating the two evidence streams can provide a broader assessment of treatment value than considering either domain in isolation.

Previous systematic reviews and network meta-analyses have predominantly compared first-line EGFR-TKI strategies in terms of efficacy, survival, response, and adverse events [10-12]. The present review therefore specifically examined randomized-trial HRQoL outcomes together with trial-informed cost-effectiveness evidence. Therefore, this systematic review aimed to synthesize two complementary evidence streams relating to first-line EGFR-TKI therapy in advanced EGFR-mutated NSCLC: randomized-trial publications reporting HRQoL outcomes and trial-informed full economic evaluations reporting cost-effectiveness outcomes. By integrating patient-reported and economic evidence, the review sought to provide a comprehensive assessment of the patient-centered and economic value of currently available first-line EGFR-TKI strategies.

## Review

Methods

Protocol Registration and Reporting Guidelines

This systematic review was conducted in accordance with the Preferred Reporting Items for Systematic Reviews and Meta-Analyses (PRISMA) 2020 statement [13]. The review protocol was prospectively registered in PROSPERO (CRD42024553180).

Eligibility Criteria

Studies were selected using the Population, Intervention, Comparator, Outcomes, and Study design framework. Eligible evidence was required to relate to randomized controlled trials evaluating first-line EGFR-TKI monotherapy in adults with advanced or recurrent EGFR-mutated NSCLC. Combination regimens involving an EGFR-TKI with chemotherapy, antibody therapy, or another systemic anticancer agent were excluded.

Two complementary types of publications were included in this review. The first comprised randomized controlled trial publications that reported HRQoL outcomes using validated patient-reported outcome instruments. The second consisted of full trial-informed economic evaluations that assessed the cost-effectiveness of first-line EGFR-TKI strategies, with the primary clinical-effectiveness data derived from the eligible randomized controlled trials.

Conference abstracts, reviews, editorials, case reports, non-comparative clinical studies, partial economic evaluations, and publications not reporting HRQoL or cost-effectiveness outcomes were excluded.

Information Sources and Search Strategy

A comprehensive literature search was performed in PubMed/MEDLINE, Embase, Scopus, and the Cochrane Library from database inception to 31 March 2026. Reference lists of eligible articles and relevant reviews were also manually searched to identify additional studies.

The search strategy combined controlled vocabulary (Medical Subject Headings and Emtree terms) with free-text keywords related to NSCLC, EGFR mutation, EGFR-TKIs, quality of life, patient-reported outcomes, cost-effectiveness, and economic evaluation. Database-specific search strategies are provided in Appendix 1.

Study Selection

After removal of duplicate records, two reviewers independently screened titles and abstracts for eligibility. Full-text articles of potentially relevant studies were subsequently assessed against the predefined inclusion and exclusion criteria. Disagreements were resolved through discussion and consensus, with consultation from a third reviewer when required. The study selection process is presented in the PRISMA 2020 flow diagram.

Data Extraction

Two reviewers independently extracted data using a standardized data extraction form. The following information was collected: study characteristics, trial name, country, patient population, intervention and comparator, HRQoL assessment instrument, HRQoL outcomes, economic model, analytical perspective, time horizon, incremental life-years, QALYs, incremental costs, ICERs, WTP thresholds, and study conclusions. Any discrepancies were resolved by consensus.

Risk of Bias and Methodological Quality Assessment

The methodological quality of randomized controlled trials reporting HRQoL outcomes was assessed independently by two reviewers using the Cochrane Risk of Bias 2 (RoB 2) tool. Studies were evaluated across five domains and classified as having low risk of bias, some concerns, or high risk of bias [14].

The methodological quality of economic evaluations was assessed using the Drummond checklist, which evaluates study design, measurement and valuation of costs and outcomes, analytical methods, uncertainty analyses, and interpretation of findings [15].

Data Synthesis

Although three HRQoL studies used the European Organisation for Research and Treatment of Cancer Quality of Life Questionnaire-Core 30 (EORTC QLQ-C30) and Quality of Life Questionnaire-Lung Cancer 13 (QLQ-LC13), differences in reported domains, assessment schedules, follow-up time points, and methods of longitudinal analysis precluded identification of sufficiently comparable effect estimates for quantitative pooling. Findings were therefore synthesized narratively. HRQoL studies were summarized according to assessment instruments and overall patient-reported outcomes, whereas economic evaluations were summarized according to model characteristics, incremental costs, QALYs, ICERs, WTP thresholds, and conclusions regarding cost-effectiveness.

Results

Studies Selected

**Figure 1:**
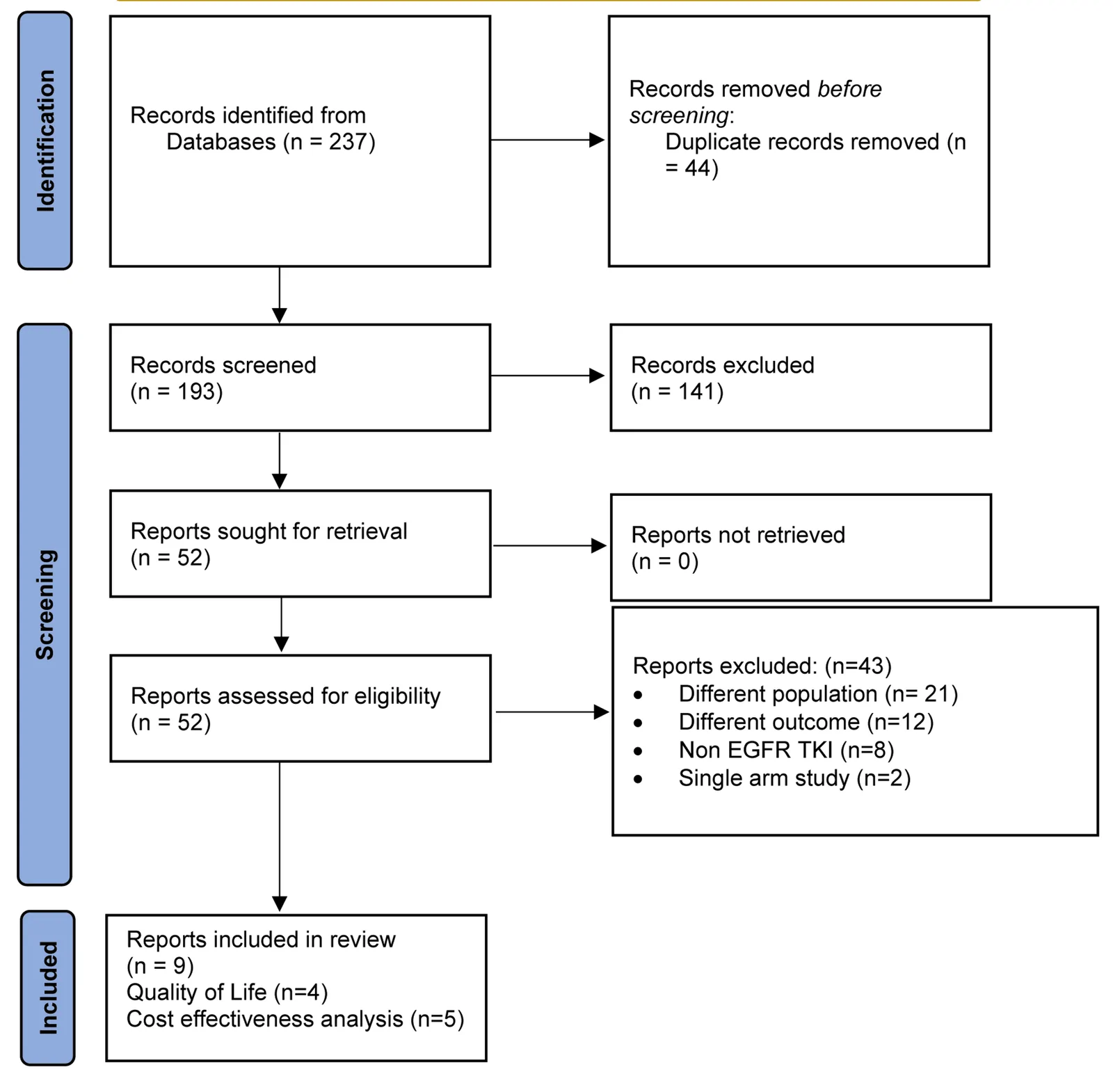
PRISMA flowchart showing number of studies at different stages of review EGFR-TKI, epidermal growth factor receptor tyrosine kinase inhibitor

The literature search identified 237 records through database searching. After removal of 44 duplicates, 193 records underwent title and abstract screening. Of these, 141 records were excluded, and 52 full-text articles were assessed for eligibility. Following full-text review, 43 articles were excluded for predefined reasons, including different study populations (n = 21), non-EGFR-TKI interventions (n = 8), absence of HRQoL or cost-effectiveness outcomes (n = 6), subset analyses (n = 6), and single-arm studies (n = 2). Nine publications linked to six pivotal randomized controlled trials were included: four trial publications reporting HRQoL outcomes and five trial-informed economic evaluations (Figure 1). Publications arising from the same underlying trial were treated as complementary reports and synthesized separately according to their respective evidence domains, thereby avoiding double-counting within the same outcome synthesis.

Characteristics of Included Studies

The characteristics of the included studies are summarized in Table 1 and Table 2. HRQoL evaluation is shown in Table 1, and economic evaluation is shown in Table 2. The nine publications included were linked to six phase III randomized controlled trials evaluating first-line EGFR-TKI therapies in advanced EGFR-mutant NSCLC.

**Table 1:** Health-related quality of life evaluation EGFR, epidermal growth factor receptor; EORTC QLQ-C30, European Organisation for Research and Treatment of Cancer Quality of Life Questionnaire-Core 30; EORTC QLQ-LC13, Quality of Life Questionnaire-Lung Cancer 13; EQ-5D, EuroQol five-dimension questionnaire; EQ-VAS, EuroQol visual analogue scale; HRQoL, health-related quality of life; NSCLC, non-small cell lung cancer

Study and main trial	Population	Intervention	Comparator	HRQoL instrument(s)
Park et al. [5], LUX-Lung 7	Treatment-naïve patients with EGFR-mutated advanced NSCLC	Afatinib	Gefitinib	EQ-5D; EQ-VAS
Paty et al. [7], ARCHER 1050	Treatment-naïve patients with EGFR-mutated advanced NSCLC	Dacomitinib	Gefitinib	EORTC QLQ-C30; EORTC QLQ-LC13
Leighl et al. [6], FLAURA	Treatment-naïve patients with EGFR-mutated advanced NSCLC	Osimertinib	Gefitinib or erlotinib	EORTC QLQ-C30; EORTC QLQ-LC13
Shi et al. [8], FURLONG	Treatment-naïve patients with EGFR-mutated advanced NSCLC	Furmonertinib	Gefitinib	EORTC QLQ-C30; EORTC QLQ-LC13

**Table 2:** Economic evaluation EGFR, epidermal growth factor receptor; NSCLC, non-small cell lung cancer

Study and main trial	Population	Intervention	Comparator	Economic model
Chouaid et al. [16], LUX-Lung 7	Treatment-naïve patients with EGFR-mutated advanced NSCLC	Afatinib	Gefitinib	Partitioned survival model
Zhang et al. [17], ARCHER 1050	Treatment-naïve patients with EGFR-mutated advanced NSCLC	Dacomitinib	Gefitinib	Partitioned survival model
Aguiar et al. [18], FLAURA	Treatment-naïve patients with EGFR-mutated advanced NSCLC	Osimertinib	Gefitinib or erlotinib	Decision-analytic model
Zhang et al. [19], AENEAS	Treatment-naïve patients with EGFR-mutated advanced NSCLC	Aumolertinib	Gefitinib	Markov model
Zhang et al. [20], MARIPOSA	Treatment-naïve patients with EGFR-mutated advanced NSCLC	Lazertinib	Osimertinib	Markov model

Health-Related Quality of Life Outcome

HRQoL outcomes are summarized in Table 3. Park et al. [5] evaluated HRQoL using the EuroQol five-dimension questionnaire (EQ-5D) and EuroQol visual analogue scale (EQ-VAS) questionnaires. HRQoL remained stable throughout treatment, with no statistically significant differences between afatinib and gefitinib in EQ-5D utility or EQ-VAS scores. Both treatment groups demonstrated maintenance of overall health status during therapy. Paty et al. [7] assessed HRQoL using the EORTC QLQ-C30 and QLQ-LC13 instruments. Global health status and functional scores remained generally stable during treatment with both dacomitinib and gefitinib. No clinically meaningful deterioration in overall HRQoL was observed despite differences in treatment-related adverse events. Leighl et al. [6] reported HRQoL using the EORTC QLQ-C30 and QLQ-LC13 questionnaires. Patients receiving osimertinib maintained global HRQoL throughout treatment, with improvements in symptom burden comparable to those observed with first-generation EGFR-TKIs. Differences in global health status between treatment groups were not statistically significant. Shi et al. [8] evaluated HRQoL using the EORTC QLQ-C30 and QLQ-LC13 questionnaires. HRQoL improved from baseline in both the furmonertinib and gefitinib groups, with no statistically significant between-group differences in global health status or functional outcomes. Overall, the available evidence consistently demonstrated that first-line EGFR-TKI therapies maintained HRQoL without clinically meaningful deterioration compared with the respective comparator treatments.

**Table 3:** Summary of health-related quality of life outcomes in randomized trials comparing second- or third-generation EGFR-TKIs with first-generation EGFR-TKIs EORTC QLQ-C30, European Organisation for Research and Treatment of Cancer Quality of Life Questionnaire Core 30; EQ-5D, EuroQol five-dimension questionnaire; EQ-VAS, EuroQol visual analogue scale; GHS/QoL, Global Health Status/Quality of Life; HRQoL, health-related quality of life; QLQ-LC13, Quality of Life Questionnaire-Lung Cancer 13 For EORTC QLQ-C30 GHS/QoL and functional scales, scores range from 0 to 100, with higher scores indicating better health-related quality of life and functioning. A change of ≥10 points is generally considered clinically meaningful.

Study	Trial	HRQoL instrument	Overall HRQoL outcome	Key HRQoL results	Authors' conclusion
Park et al. [5]	LUX-Lung 7	EQ-5D, EQ-VAS	HRQoL remained comparable between treatment groups.	Baseline EQ-5D utility: 0.72 ± 0.26 vs 0.73 ± 0.25; post-baseline adjusted mean: 0.77 vs 0.80 (P = 0.14). Baseline EQ-VAS: 69.7 ± 19.3 vs 71.2 ± 17.0; post-baseline adjusted mean: 74.5 vs 76.0 (P = 0.203).	Afatinib maintained overall HRQoL comparable to gefitinib, with no significant differences in overall health status.
Paty et al. [7]	ARCHER 1050	EORTC QLQ-C30, QLQ-LC13	Global QoL remained stable with dacomitinib and showed slight, non-clinically meaningful improvement with gefitinib.	GHS/QoL scores ranged from 61.5 (cycle 30) to 69.8 (cycle 28) with dacomitinib and 68.5 (cycle 17) to 73.1 (cycle 28) with gefitinib. No clinically meaningful (≥10-point) changes in Global QoL or physical functioning were observed.	Overall HRQoL was maintained throughout dacomitinib treatment, with no clinically meaningful deterioration in global QoL or physical functioning.
Leighl et al. [6]	FLAURA	EORTC QLQ-C30, QLQ-LC13	Overall HRQoL was maintained with osimertinib.	Baseline GHS/QoL: 62.5 ± 23.2 vs 58.8 ± 22.8; adjusted mean change from baseline: treatment difference: 2.06 (95% CI -0.85 to 4.98; P = 0.165).	Osimertinib maintained overall HRQoL while improving several functional and symptom domains compared with standard EGFR-TKIs.
Shi et al. [8]	FURLONG	EORTC QLQ-C30, QLQ-LC13	Overall HRQoL improved in both groups and was maintained with furmonertinib.	Baseline HRQoL: 64.4 ± 21.0 vs 63.7 ± 20.9. Mean HRQoL change from baseline: 3.80 (95% CI 1.76-5.85) with furmonertinib vs 2.60 (95% CI 0.51-4.69) with gefitinib; adjusted between-group difference 1.21 (95% CI -1.71 to 4.13; P = 0.417). No clinically relevant (≥10-point) between-group difference in overall HRQoL was observed.	Furmonertinib maintained overall HRQoL while improving patient-reported outcomes compared with gefitinib.

Cost-Effectiveness Analyses

Five full economic evaluations were included (Table 4). Chouaid et al. [16] reported that afatinib was cost-effective compared with gefitinib in the French healthcare setting. Economic evaluations based on the FLAURA and ARCHER 1050 trials concluded that osimertinib and dacomitinib, respectively, were not cost-effective because their ICERs exceeded the study-specific WTP thresholds [17,18]. Zhang et al. [19] demonstrated that aumolertinib was cost-effective compared with gefitinib in China. Similarly, the economic evaluation based on the MARIPOSA trial found that lazertinib was not cost-effective because its ICER exceeded the study-specific WTP threshold [20].

**Table 4:** Summary of economic evaluations of first-line EGFR-TKI therapies in advanced EGFR-mutated NSCLC EGFR-TKI, epidermal growth factor receptor tyrosine kinase inhibitor; GDP, gross domestic product; ICER, incremental cost-effectiveness ratio; LY, life-year; NR, not reported; QALY, quality-adjusted life-year; WTP, willingness-to-pay

Study	Trial	Country	Economic model	Time horizon	Incremental LY	Incremental QALY	Incremental cost	ICER (cost/QALY gained)	WTP threshold	Cost-effective?
Chouaid et al. (2017) [16]	LUX-Lung 7	France	Partitioned survival model	10 years	0.249	0.170	€7,697	€45,211/QALY	€70,000/QALY	Yes
Aguiar et al. (2018) [18]	FLAURA	USA/Brazil	Three-state decision-analytic model	10 years	1.01	0.594	Not reported as a single overall value	USA- US $225,000/QALY Brazil- US $172,000/QALY	WHO-recommended threshold (≈3× GDP per capita)	No
Zhang et al. (2021) [17]	ARCHER 1050	USA	Partitioned survival model	10 years	1.298	0.706	US$232,359.32	US$329,121/QALY	US$100,000/QALY	No
Zhang et al. (2024) [19]	AENEAS	China	Three-state Markov model	20 years	0.941	0.692	US$18,855.55	US$27,272.29/QALY	Approximately US$37,000/QALY (3× GDP per capita)	Yes
Zhang et al. (2025) [20]	MARIPOSA	China	Three-state Markov model	5 years	NR	0.710	US$224,248	US$315,640/QALY	Approximately US$36,887/QALY (3× GDP per capita)	No

The included studies used partitioned survival or three-state Markov models with time horizons ranging from five years to lifetime. Considerable variation was observed in analytical perspective, healthcare setting, model assumptions, and WTP thresholds, precluding direct comparison of economic outcomes across studies.

Risk-of-Bias Assessment

The risk-of-bias assessment is presented in Table 5. Two studies (FLAURA and FURLONG) were judged to have an overall low risk of bias, whereas LUX-Lung 7 and ARCHER 1050 were assessed as having some concerns, primarily because of their open-label design. No study was considered to have a high overall risk of bias.

**Table 5:** Risk of Bias (RoB 2) assessment of included HRQoL studies HRQoL, health-related quality of life

Study	Randomization process	Deviations from intended interventions	Missing outcome data	Measurement of outcome	Selection of reported result	Overall RoB
Park et al. (LUX-Lung 7) [5]	Low	Some concerns	Low	Low	Low	Some concerns
Paty et al. (ARCHER 1050) [7]	Low	Some concerns	Low	Low	Low	Some concerns
Leighl et al. (FLAURA) [6]	Low	Low	Low	Low	Low	Low
Shi et al. (FURLONG) [8]	Low	Low	Low	Low	Low	Low

Methodological Quality of Economic Evaluations

The methodological assessment of economic evaluations using the Drummond checklist is summarized in Appendix 2. All five studies satisfied the major methodological criteria, including a clearly defined study question, appropriate comparators, valid effectiveness data, comprehensive measurement of costs and outcomes, incremental analysis, and sensitivity analyses. Overall, the included economic evaluations were considered to be of high methodological quality.

Discussion

This systematic review synthesized randomized-trial evidence on HRQoL together with trial-informed economic evaluations of first-line EGFR-TKIs in advanced EGFR-mutated NSCLC. Across the included HRQoL studies, global health status and functional outcomes were generally maintained during treatment, with no consistent evidence of clinically meaningful between-group differences demonstrated. In contrast, cost-effectiveness findings varied substantially across healthcare systems and were influenced by drug prices, analytical perspectives, model assumptions, and WTP thresholds.

Across LUX-Lung 7, ARCHER 1050, FLAURA, and FURLONG, overall HRQoL remained broadly stable or improved from baseline in both intervention and comparator groups [5-8]. This is clinically relevant because EGFR-TKIs are usually administered continuously for prolonged periods. Although individual agents differed in adverse-event profiles, these differences did not consistently translate into clinically meaningful deterioration in global HRQoL. Common toxicities may be partly mitigated by supportive care, dose reduction, or temporary treatment interruption, while effective disease control may offset some of their negative effects. However, global HRQoL measures may not fully capture treatment-specific burdens, and differences in individual symptoms or functional domains were reported in some trials.

These findings support continued incorporation of patient-reported outcomes into randomized trials and treatment decisions. Progression-free and overall survival remain central measures of efficacy, but HRQoL provides complementary information about symptoms, functioning, and the patient’s experience of treatment.

The economic evaluations showed greater variability. Afatinib and aumolertinib were considered cost-effective in the settings examined, whereas osimertinib, dacomitinib, and lazertinib exceeded the WTP thresholds used in the respective analyses [16-20]. These results should not be interpreted as universal rankings of treatment value. Cost-effectiveness depends on local drug prices, subsequent treatment costs, reimbursement arrangements, utility assumptions, survival extrapolation, and the threshold adopted by each healthcare system. Drug acquisition and subsequent-treatment costs influence incremental costs, whereas utility estimates and survival extrapolation affect projected QALYs. Differences in these inputs may substantially alter ICERs, while healthcare-system-specific WTP thresholds determine whether an ICER is considered cost-effective. Comparative modelling across the United Kingdom and China similarly demonstrated that the economic value of first-line EGFR-TKI strategies may differ between countries [12].

Previous systematic reviews and network meta-analyses have mainly compared first-line EGFR-TKIs according to progression-free survival, overall survival, response, and adverse events [9-11]. Individual randomized trials have reported HRQoL outcomes [5-8], whereas economic evaluations have generally assessed selected drugs and national healthcare settings [12,16-20]. Published syntheses integrating randomized-trial HRQoL findings with trial-informed cost-effectiveness evidence appear limited. The present review therefore provides a broader assessment of treatment value by examining patient-reported and economic outcomes within a single systematic-review framework. More recent first-line combination strategies, including osimertinib plus chemotherapy and EGFR-directed antibody/TKI combinations, may alter the balance between clinical benefit, toxicity, HRQoL, and treatment costs. As these combination strategies were outside the prespecified monotherapy scope of the present review, their patient-reported and economic outcomes warrant separate evaluation.

Limitations

Several limitations should be acknowledged. Only four eligible publications reported randomized-trial HRQoL outcomes, limiting comparisons across all available EGFR-TKIs. Heterogeneity in HRQoL instruments, assessment schedules, economic-model structures, analytical perspectives, time horizons, currencies, and WTP thresholds precluded quantitative synthesis and limited direct comparison across studies. Economic findings were dependent on country-specific inputs and modelling assumptions, including extrapolated survival, externally sourced utility values, and subsequent-treatment pathways. Despite the comprehensive database search, the possibility that eligible studies were missed cannot be excluded, particularly given the rapidly evolving EGFR-mutated NSCLC treatment landscape. Further, clinical trial registries and grey literature were not systematically searched, and potentially relevant unpublished or ongoing studies may therefore have been missed. Publication bias could not be formally assessed because of the small and heterogeneous evidence base. In addition, trial-derived HRQoL findings may not fully reflect routine clinical practice, particularly among patients with poorer performance status or greater comorbidity.

## Conclusions

Available randomized-trial evidence generally demonstrated maintenance of HRQoL during first-line EGFR-TKI treatment, with no consistent clinically meaningful between-group differences demonstrated; however, the limited evidence base does not establish equivalence between individual EGFR-TKIs. In contrast, cost-effectiveness varied markedly across healthcare systems and appeared to be largely driven by drug acquisition costs and country-specific WTP thresholds. These findings were context-specific and depended on local drug prices, modelling assumptions, healthcare-system characteristics, and WTP thresholds; therefore, economic conclusions from one setting should not be directly generalized to another. Thus, the principal value distinction among first-line EGFR-TKIs may lie less in global HRQoL and more in whether additional clinical benefit is considered affordable within the local healthcare setting.

## Appendices

Appendix 1 

**Table 6:** Search strategy

Database	Search string
PubMed	(("non-small cell lung cancer"[Title/Abstract] OR NSCLC[Title/Abstract]) AND (EGFR[Title/Abstract]) AND ("quality of life" OR "patient-reported outcomes" OR PRO OR HRQoL OR "cost-effectiveness" OR "economic evaluation"))
Embase	(('non-small cell lung cancer':ti,ab OR NSCLC:ti,ab) AND (EGFR:ti,ab) AND ('quality of life' OR 'patient-reported outcomes' OR PRO OR HRQoL OR 'cost-effectiveness' OR 'economic evaluation'))
Scopus	TITLE-ABS (("non-small cell lung cancer" OR NSCLC) AND (EGFR) AND ("quality of life" OR "patient-reported outcomes" OR PRO OR HRQoL OR "cost-effectiveness" OR "economic evaluation"))
Cochrane CENTRAL	(("non-small cell lung cancer" OR NSCLC) AND (EGFR) AND ("quality of life" OR "patient-reported outcomes" OR PRO OR HRQoL OR "cost-effectiveness" OR "economic evaluation"))

Appendix 2 

**Table 7:** Methodological quality assessment of included cost-effectiveness studies using the Drummond checklist The Zhang et al. [20] (MARIPOSA) economic evaluation assessed two intervention strategies against osimertinib. Only the lazertinib monotherapy comparison was included in the review’s economic results to maintain consistency with the prespecified eligibility criterion of first-line EGFR-TKI therapies.

Drummond criterion	Chouaid et al. (2017) LUX-Lung 7 [16]	Aguiar et al. (2018) FLAURA [18]	Zhang et al. (2021) ARCHER 1050 [17]	Zhang et al. (2024) AENEAS [19]	Zhang et al. (2025) MARIPOSA [20]
1. Was a well-defined study question stated?	Yes. Compared afatinib with gefitinib as first-line therapy in EGFR-mutated NSCLC from the French healthcare perspective.	Yes. Evaluated first-line osimertinib vs. earlier-generation EGFR-TKIs from US and Brazilian payer perspectives.	Yes. Evaluated dacomitinib vs. gefitinib from a US payer perspective.	Yes. Evaluated aumolertinib vs. gefitinib from the Chinese healthcare perspective.	Yes. Evaluated lazertinib and amivantamab-lazertinib vs. osimertinib from the Chinese healthcare perspective.
2. Were the competing alternatives described?	Yes. Intervention, comparator, treatment sequence, and model structure clearly described.	Yes. Comparator strategies and subsequent treatment pathways explicitly defined.	Yes. Treatment strategies and post-progression management described.	Yes. Both first-line and subsequent therapies clearly specified.	Yes. Three competing first-line strategies were explicitly modelled.
3. Was effectiveness established?	Yes. Clinical efficacy obtained directly from the randomized LUX-Lung 7 trial.	Yes. Used FLAURA trial data supplemented by published meta-analysis.	Yes. Clinical inputs derived from ARCHER 1050 survival data.	Yes. Clinical outcomes obtained from the AENEAS phase III trial.	Yes. Clinical efficacy derived from the MARIPOSA randomized trial.
4. Were all important costs identified?	Yes. Drug acquisition, disease management, adverse events, and terminal care included.	Yes. Drug, monitoring, adverse events, supportive care and post-progression costs included.	Yes. Direct medical costs, subsequent treatment, and adverse-event costs considered.	Yes. Direct medical costs, including drugs, monitoring, adverse events, and subsequent therapy, included.	Yes. Drug, administration, monitoring, adverse-event, and disease-management costs incorporated.
5. Were costs measured accurately?	Yes. Resource utilization derived from the clinical trial and expert opinion.	Yes. Resource use based on FLAURA and payer cost schedules.	Yes. Cost inputs obtained from published US sources and standard treatment pathways.	Yes. Resource use based on Chinese provincial reimbursement schedules and trial protocol.	Yes. Cost inputs obtained from Chinese pricing schedules and published sources.
6. Were costs valued credibly?	Yes. French national tariffs and literature sources used.	Yes. Official US Medicare and Brazilian payer prices applied.	Yes. US drug pricing and published cost sources employed.	Yes. National/provincial Chinese cost data used.	Yes. Chinese healthcare pricing data used consistently.
7. Were outcomes measured and valued appropriately?	Yes. LYs, QALYs, and ICERs estimated using validated utilities.	Yes. QALYs estimated using published utilities within a decision-analytic model.	Yes. LYs and QALYs estimated using published utility values.	Yes. LYs and QALYs estimated using published utility values and Markov modelling.	Yes. QALYs estimated using validated utilities within a Markov model.
8. Was discounting applied appropriately?	Yes. Future costs and outcomes discounted.	Yes. Long-term costs and outcomes discounted over a 10-year horizon.	Yes. Annual 3% discount rate applied.	Yes. Costs and utilities discounted according to Chinese pharmacoeconomic guidance.	Yes. Annual 3% discounting applied.
9. Was uncertainty adequately explored?	Yes. Deterministic and probabilistic sensitivity analyses performed.	Yes. Extensive sensitivity analyses evaluating drug prices and survival assumptions.	Yes. One-way and probabilistic sensitivity analyses conducted.	Yes. One-way, probabilistic, and scenario analyses undertaken.	Yes. One-way, probabilistic, and scenario analyses performed.
10. Were results presented and discussed appropriately?	Yes. Results, limitations, and policy implications discussed.	Yes. Results interpreted within payer-specific contexts and study limitations acknowledged.	Yes. Findings, limitations, and implications clearly discussed.	Yes. Interpretation included threshold analysis, limitations, and implications for Chinese practice.	Yes. Results discussed together with sensitivity analyses and study limitations.
Overall methodological quality	High	High	High	High	High
